# Iron to Gas: Versatile Multiport Flow-Column Revealed Extremely High Corrosion Potential by Methanogen-Induced Microbiologically Influenced Corrosion (Mi-MIC)

**DOI:** 10.3389/fmicb.2020.00527

**Published:** 2020-03-31

**Authors:** Biwen Annie An, Sherin Kleinbub, Ozlem Ozcan, Andrea Koerdt

**Affiliations:** Bundesanstalt für Materialforschung und -prüfung, Berlin, Germany

**Keywords:** microbiologically influenced corrosion, methanogen, methane, biocorrosion, flow system, modeling, multiport

## Abstract

Currently, sulfate-reducing bacteria (SRB) is regarded as the main culprit of microbiologically influenced corrosion (MIC), mainly due to the low reported corrosion rates of other microorganisms. For example, the highest reported corrosion rate for methanogens is 0.065 mm/yr. However, by investigating methanogen-induced microbiologically influenced corrosion (Mi-MIC) using an in-house developed versatile multiport flow test column, extremely high corrosion rates were observed. We analyzed a large set of carbon steel beads, which were sectionally embedded into the test columns as substrates for iron-utilizing methanogen *Methanobacterium* IM1. After 14 days of operation using glass beads as fillers for section separation, the highest average corrosion rate of *Methanobacterium* IM1 was 0.2 mm/yr, which doubled that of *Desulfovibrio ferrophilus* IS5 and *Desulfovibrio alaskensis* 16109 investigated at the same conditions. At the most corroded region, nearly 80% of the beads lost 1% of their initial weight (fast-corrosion), resulting in an average corrosion rate of 0.2 mm/yr for *Methanobacterium* IM1-treated columns. When sand was used as filler material to mimic sediment conditions, average corrosion rates for *Methanobacterium* IM1 increased to 0.3 mm/yr (maximum 0.52 mm/yr) with over 83% of the beads having corrosion rates above 0.3 mm/yr. Scanning electron images of metal coupons extracted from the column showed methanogenic cells were clustered close to the metal surface. *Methanobacterium* IM1 is a hydrogenotrophic methanogen with higher affinity to metal than H_2_. Unlike SRB, *Methanobacterium* IM1 is not restricted to the availability of sulfate concentration in the environment. Thus, the use of the multiport flow column provided a new insight on the corrosion potential of methanogens, particularly in dynamic conditions, that offers new opportunities for monitoring and development of mitigation strategies. Overall, this study shows (1) under certain conditions methanogenic archaea can cause higher corrosion than SRB, (2) specific quantifications, i.e., maximum, average, and minimum corrosion rates can be determined, and (3) that spatial statistical evaluations of MIC can be carried out.

## Introduction

Steel is the most used metal on Earth due to its material properties, such as high strength and formability, as well as low production costs (Enning and Garrelfs, 2014). Production of steel has been increasing in the past decade and nearly 1.7 billion metric tons are produced annually (U.S. Department of Commerce-International Trade Administration, 2018). Several industries rely heavily on the use of steel, i.e., water transport pipelines, cooling circuits, storage tanks, parts of production facilities, or petroleum production wells. One serious challenge for the extensively used steel infrastructure is corrosion monitoring and control. In 2013, the global cost of corrosion was $2.5 trillion USD (3.4% GDP) (Koch et al., 2016). It is essential to develop effective monitoring and preventative methods to minimize economic and environmental impacts. In contrast to abiotic corrosion, which are relatively straightforward to predict and mitigate, microbiologically influenced corrosion (MIC) is highly unpredictable due to the diversity of microbial communities involved and the type of corrosion that microorganisms cause. Microorganisms cause the so-called pitting corrosion, a localized form of corrosion where the initiation mechanisms are not yet fully understood (Kakooei et al., 2012; Enning and Garrelfs, 2014; Pinnock et al., 2018). Currently, there are two main types of MIC mechanisms identified: indirect microbiologically-influenced corrosion and direct microbiologically-influenced corrosion (Enning and Garrelfs, 2014; Jia et al., 2019). Indirect corrosion through the production of corrosive metabolites or through electron mediators (Al Abbas et al., 2013; Pinnock et al., 2018; Jia et al., 2019), and direct uptake of electrons from the metal surface using cytochrome or pili, hereby leading to metal dissolution are well-identified for sulfate-reducing bacteria (SRB) (Kakooei et al., 2012; Enning and Garrelfs, 2014; Pinnock et al., 2018; Jia et al., 2019). Reduction of sulfate by SRB using either organic acids (Eq. 1) or iron (Eq. 2) as electron donor results in the production of sulfide or sulfite that forms a corrosive cell (cathode) on the metal surface (anode) (Enning and Garrelfs, 2014; Rémazeilles et al., 2017).

(1) $${\text{SO}}_{4}^{2-}+{\text{C}}_{2}\,{\text{H}}_{3}\,{\text{O}}_{2}^{-}\,\left(\text{acetate}\right)\to {\text{HS}}^{-}+2\,H\,C\,O_{3}^{-}$$

(2) $$4\,F\,e^{\text{o}}+{\text{SO}}_{4}^{2-}+3\,H\,C\,O_{3}^{-}+5\,H^{+}\to \text{FeS}+3\,F\,e\,C\,O_{3}+4\,H_{2}\,\text{O}$$

In addition to SRB, acetogens, iron-reducers/oxidizers, sulfide-oxidizers and methanogenic archaea (MA) are also corrosive (Dinh et al., 2004; Mori et al., 2010; Park et al., 2011; Sun et al., 2014; Kato et al., 2015; Mand et al., 2016). MA can metabolize limited substrates for growth, including H_2_/CO_2_, acetate, methanol and methylamines (Youssef et al., 2009; Tucker et al., 2015). Certain strains of MA have also adapted to direct electron withdrawal from steel for methanogenesis (methanogen-induced microbiologically-influenced corrosion or Mi-MIC) (Dinh et al., 2004; Lienemann et al., 2018; Tsurumaru et al., 2018), though the specific mechanisms remain largely unknown. Recently, the corrosive *Methanococcus maripaludis* OS7 was identified to exhibit a corrosive genomic island or MIC-island (Tsurumaru et al., 2018). The MIC-island is an approximately 10 kb gene segment that encodes for a hydrogenase and a transport system that is most likely responsible for the secretion of the hydrogenase. It was proposed that the hydrogenase can attach to the metal surface and use the electrons from the metal oxidation for hydrogen production. Produced hydrogen is then utilized as an electron donor for methanogenesis. Currently, such a mechanism is only identified for a few *Methanococcus* strains and not conclusive for all corrosive methanogenic strains. Members of *Methanobacteriales* (Dinh et al., 2004) and the recently discovered Baltic-*Methanosarcina* (Palacios et al., 2019) are shown to exhibit higher iron-affinity than H_2_ (Beese-Vasbender et al., 2015; Palacios et al., 2019). However, Mi-MIC received little recognition largely due to the reported low corrosion rates (between 0.02 and 0.065 mm/yr) (Daniels et al., 1987; Mori et al., 2010; Liang et al., 2014). In comparison, highly corrosive SRB can reach corrosion rates between 0.31 and 0.71 mm/yr (Enning et al., 2012). One explanation for the low corrosion rates of methanogens is the assumed production of the non-conductive corrosion product siderite (Eqs 3 and 4; FeCO_3_) (Kip et al., 2017). However, the formation of siderite as the only corrosion product remains questionable. The stability of siderite strictly depends upon the supersaturation level (S; Eq. 5), which positively correlates with the concentrations of ferrous and carbonate ions in the solution under stable conditions and inversely relates to the solubility factor (K_sp_; Eq. 5). Several factors, including salinity, pH, temperature and pressure, will affect K_sp_, limiting the formation of the stable crystallized structure of siderite (Joshi, 2015; Barker et al., 2018).

(3) $$8\,H^{+}+4\,F\,e^{\text{o}}+{\text{CO}}_{2}\to {\text{CH}}_{4}+4\,F\,e^{2+}+2\,H_{2}\,\text{O}$$

(4) $${\text{Fe}}^{2+}+{\text{HCO}}_{3}^{-}\to {\text{FeCO}}_{3}+{\text{CO}}_{2}+{\text{H}}_{2}\,\text{O}$$

(5) $$\text{S}=\frac{\left[F\,e^{2+}\right]\,\left[C\,O_{3}^{2-}\right]}{K_{s\,p}}$$

Under abiotic CO_2_-rich environments, i.e., oil and gas pipelines, semi-conductive magnetite (Fe_3_O_4_) and chukanovite (Fe_2_(OH)_2_CO_3_) could also be formed (Joshi, 2015; Barker et al., 2018), due to the inability of siderite reaching the supersaturation level. In the presence of brine solutions, additional minerals such as MgCO_3_ and CaCO_3_ are possible corrosion products (Barker et al., 2018; in ‘t Zandt et al., 2019) and changing the state of the siderite crystal structure. Furthermore, many of the isolated corrosive MA are marine microorganisms that require a salinity of 0.6 M_eq of NaCl_ for growth, at this salinity the K_sp_ would decrease resulting in an increased siderite solubility (Barker et al., 2018). However, all experiments reporting on MA corrosion were conducted in stationary laboratory-controlled settings, this allowed an undisturbed formation of siderite and are unrepresentative of natural conditions.

Therefore, a standard environmental-simulated model is required to fully understand methanogenic corrosion. Thus, we developed a versatile multiport flow system to bridge between the field testing and stationary laboratory experiments. Here we report the efficacy of the system on an iron-utilizing methanogenic strain, in comparison to well-studied SRB strains under both flow and stationary conditions. Corrosion rate distributions and ideal testing conditions for both methanogens and SRB are reported here.

## Materials and Methods

### Strains, Media, and Culturing Conditions

M*ethanobacterium*-affiliated IM1 (Dinh et al., 2004) isolate was obtained from Dr. Friedrich Widdel (Max Planck Institute for Marine Microbiology, Germany), *Desulfovibrio ferrophilus* IS5 and *Desulfovibrio alaskensis* 16109 were purchased from DSMZ culture collection (DSMZ, Germany). Cultures (50 mL in 120 mL bottle) were maintained in anoxic artificial seawater medium (ASW; 26.37 g/L NaCl, 0.6 g/L KCl, 11.18 g/L MgCl_2_⋅6H_2_O, 1.47 g/L CaCl_2_⋅2H_2_O, 0.66 g/L KH_2_PO_4_, and 0.25 g/L NH_4_Cl) (Widdel and Bak, 1992), supplemented with selenite-tungstate, vitamin, trace element (Widdel and Bak, 1992), riboflavin (0.664 μM), lipoic acid (0.727 μM), folic acid (0.906 μM), and sodium acetate (1 mM) (Dinh et al., 2004). Sodium sulfide (1 mM) and cysteine (1 mM) were added as reducing agents (Widdel and Bak, 1992). Medium was buffered with CO_2_/HCO_3_ (pH 7.2–7.3). Cultures were closed with butyl rubber stoppers and aluminum caps, under N_2_/CO_2_ (80%:20% v/v) at 30°C. Sterilized steel coupons (99.5% Fe^o^, 0.3% Mn, 0.08% C, 0.04% P, 0.8 cm × 0.8 cm × 0.1 cm, Goodfellow GmbH, Germany), carbon steel beads (AISI 1010, Ø = 0.238 cm, Rockwell hardness C60 to C67, grade 1000, 99.18–99.62% Fe^o^, 0.30–0.60% Mn, 0.08–0.13% C, 55.22 ± 0.026 mg, Simply bearings, England) (Pinnock et al., 2018) or lactate (20 mM) served as electron donors during incubation with (*D. ferrophilus* IS5, *D. alaskensis* 16109) or without (*Methanobacterium* IM1), 10 mM sodium sulfate as electron acceptor for SRB. Once the cultures are fully grown, 30–35% of the cultures were used to inoculate the columns.

### Stationary Batch Culture Experiments

Batch culture experiments were conducted in 120 mL serum bottles filled with 50 mL ASW medium and 70 mL headspace (N_2_/CO_2_, 80%:20% v/v). Medium for *D. alaskensis* 16109 was supplemented with 10 mM Na_2_SO_4_ and 20 mM lactate, medium for *D. ferrophilus* IS5 was supplemented with 10 mM SO_4_. Additionally, a metal coupon (99.5% Fe^o^, 0.3% Mn, 0.08% C, 0.04% P, 0.8 cm × 0.8 cm × 0.1 cm, Goodfellow GmbH, Germany) was placed in each bottle, prior polished with sandpaper (grit 320) and treated with acid according to the NACE protocol SP0775-2013 (NACE, 2013). All biological experiments were conducted in triplicates (abiotic controls were duplicated) at 30°C for 14 days. To determine the growth of microorganisms, headspace methane and dissolved sulfide concentrations were used as indicators. Headspace methane was measured using a custom-built Agilent 7890B equipped with a thermal conductivity detector (Teckso GmBH, Germany). A Msieve 5A column (Teckso, GmBH; 30-cm length, 1.0-mm i.d., and 80/100 mesh) was used with argon as the carrier gas with a set flow at 25 mL/min. The GC was controlled and automated with the PAL3 and Chronos software (Teckso, GmBH, Germany). The detection limit for methane is 20 ppm. The dissolved hydrogen sulfide concentration in the medium and enrichments of *D. alaskensis* 16109 and *D. ferrophilus* IS5, were determined using the diamine or methylene blue method (Trüper and Schlegel, 1964; Xue and Voordouw, 2015; An et al., 2017).

### Multiport Flow-Column Set-Up

**FIGURE 1:**
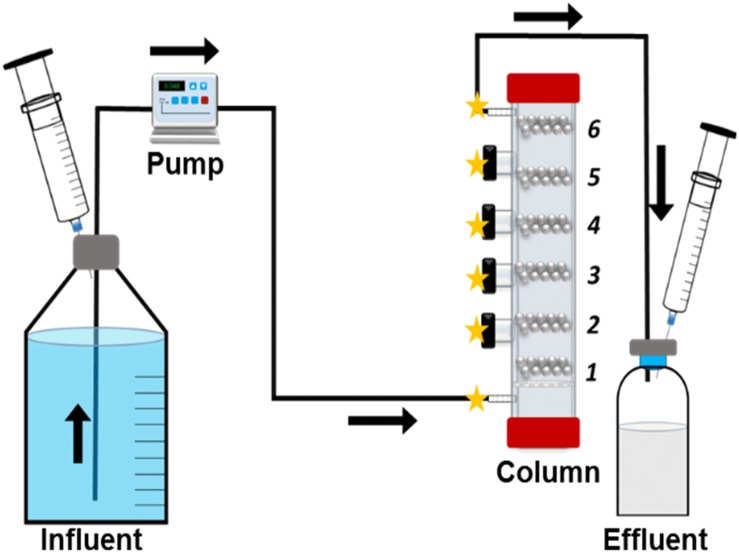
Schematic overview of the flow through system. Column sections (1–6) and possible sampling points (stars) are identified.

A schematic illustration of the flow through system is presented in Figure 1. Glass columns were constructed with four side ports (Figure 1) (Glasgerätebau Ochs GmbH, Germany). The entire system is designed to be operated under anaerobic conditions, as rubber stopper protected side ports allow anaerobic samplings and injections, i.e., biocides. At the bottom of the column, a glass filter is placed to prevent back flow into the medium reservoir to prevent clogging (Figure 1). Column is divided into 6 sections (Figure 1), section 1 is the influent region and section 6 is the effluent region. The column is designed as a one-directional flow system from bottom–up, allowing the methane gas to be collected at the effluent site and to ensure an even media flow through the entire length of the column (Figure 1). Each section is filled with 20 sterile carbon steel beads. The carbon steel beads are extremely uniform with minimal weight differences (55.22 ± 0.026 mg) and high surface area to volume ratio (25 mm^–1^) (Voordouw et al., 2016). The uniformity of the carbon steel beads plays an important role in illustrating localized corrosion and calculation of the corrosion rate distributions at different sections of the column. The beads were pre-treated with acid according to the NACE protocol SP0775- 2013 (NACE, 2013). Sections of the column were separated with glass beads (Ø = 0.3 cm, Glasgerätebau Ochs GmbH, Germany) or sand (0.09–0.20 mm particle size, DIN EN 60312-1:2017, Normen Sand, Germany). In addition, one pre-treated coupon was inserted at section 1, for surface analyses (99.5% Fe^o^, 0.3% Mn, 0.08% C, 0.04% P, 0.8 cm × 0.8 cm × 0.1 cm, Goodfellow GmbH, Germany). Pore volume (PV) of the columns was calculated using the weight differences between the dry packed column and the anaerobic medium-flooded wet columns (PV = 32.6 ± 1.8 mL for sand and PV = 36.1 ± 2.4 mL for glass beads). Cultures (0.5 PV) were directly incubated into the media-flooded columns through the influent port (section 1; Figure 1). Columns were sealed and incubated at 30°C for 84 h or 7 days without flow. Post-incubation, fresh anaerobic ASW media were continuously injected into the columns. For *D. alaskensis* 16109 and *D. ferrophilus D. ferrophilus* IS5, media were additionally supplemented with 20 mM lactate and 4 mM sulfate or 4 mM sulfate only, respectively. All biological experiments were conducted in triplicates (abiotic controls were duplicated) at 30°C. The system flow rate was 0.048 mL/min or 60 mL/24 h, converting to ∼2 PV/day. All effluents from the columns were collected in individually sealed serum bottles (Figure 1) for methane measurements. Periodic sulfide measurements were conducted by collecting samples from individual sections of the column (Figure 1). At the end of the experiment, columns were taken apart by section and cleaned using NACE protocol SP0775- 2013 (Haroon et al., 2013; Pinnock et al., 2018). Beads were weighed before and after the experiment by section using a Sartorius analytical scale (Sartorius AG, Germany) to calculate the individual corrosion rate (CR). Additional modifications of the system can be achieved by using different materials, i.e., stainless steel, or a chemostat can be added to allow continuous injection of fresh cultures.

### Corrosion Rate Calculation and Surface Analyses

The general corrosion rates of individual bead and coupon from stationary and flow-through systems were calculated using the weight loss (ΔW in g) method. The corrosion rate CR (mm/yr) was calculated as:

(6) CR=87,600×Δ⁢W/(D*A*T)

Where D is the density of the steel (7.85 g/cm^3^), A is the surface area of the coupon (1.6 cm^2^) or of the beads (0.178 cm^2^) and T is the operation time in hr. The factor 87,600 converts the measured corrosion rate from cm/hr to mm/yr.

Coupons extracted from the column were first fixated with 2.5% glutaraldehyde overnight at 4°C, followed by ethanol dehydration (30, 50, 70, 80, 90% and absolute). Coupons were dried with N_2_ gas and placed in vacuum chamber prior to SEM. Scanning electron microscope (SEM) images were acquired and analyzed using a Zeiss EVO MA10 microscope with a tungsten hairpin cathode. Images were obtained using an acceleration voltage of 7 kV.

## Results

### Stationary Batch Culture Experiments

Stationary enrichments with iron coupons were used to compare corrosion rates with flow system. To confirm the growths of methanogen and SRB, methane and hydrogen sulfide concentrations were measured, respectively. Increased methane concentrations of *Methanobacterium* IM1 (highest 1.8 ± 0.38 mM; Supplementary Figure S1) confirmed the growths of all biological replicates. After 14 days, the aqueous hydrogen sulfide concentrations for *D. alaskensis* 16109 and *D. ferrophilus* IS5 cultures were 3.8 ± 0.98 and 0.95 ± 0.16 mM, respectively (Supplementary Figure S2). In the stationary batch culture experiments, the highest corrosion rate was observed for *D. ferrophilus* IS5, which was 0.35 ± 0.11 mm/yr, 3.6-fold higher than the flow system corrosion rate (Table 1). However, corrosion rates for *D. alaskensis* 16109 (0.034 ± 0.005 mm/yr) and *Methanobacterium* IM1 (0.15 ± 0.019 mm/yr) were significantly lower than that of the flow system. In particularly, the corrosion rate of *D. alaskensis* 16109 was only slightly higher than that of the abiotic control, which was 0.022 ± 0.002 mm/yr. Based on the results of the stationary cultures, *D. ferrophilus* IS5 is 10 times more corrosive than *D. alaskensis* 16109 and 2.3 times more corrosive than *Methanobacterium* IM1 (Table 1). However, distribution and determination of maximum/minimal corrosion rates could not be calculated (Table 1 and Supplementary Table S1).

**TABLE 1:** Comparison of corrosion rates and distribution between flow-through system (section 1 only) and stationary cultures. Sulfate reducing bacteria (*D. alaskensis* and *D. ferrophilus* IS5) are compared against methanogen (*Methanobacterium* IM1).

		**Flow through system**											
		**Glass-filled**					**Sand-filled**			**Stationary cultures**			
				
	**Column section**	Control*	**16109^1^**	**IS5^1^**	**IM1^1^ set 1**	**IM1^1^ set 2**	**Control**	**16109^2^**	**IM1^2^**	**Control**	**16109**	**IS5**	**IM1**
Total^3^_fast_ (%^4^)	1	10	28	35	**88**	**78**	30	55	**83**	N/A			
CR_Fast_ (mm/yr)	1	0.12	0.16	0.15	**0.22**	**0.21**	0.12	0.17	**0.36**				
Total^5^_Slow_ (%)	1	90	72	65	12	22	70	45	17				
CR_Slow_ (mm/yr)	1	0.04	0.08	0.07	0.05	0.06	0.08	0.07	0.06				
CR_Fast_/CR_Slow_	1	2.6	2.0	2.2	**4.1**	**3.6**	1.6	2.3	**6.4**				
CR_Avg_ (mm/yr)	**1**	**0.05**	**0.10**	**0.10**	**0.20**	**0.17**	**0.06**	**0.13**	**0.31**	**0.02**	**0.03**	**0.35**	**0.15**

^1^Columns were incubated for 84 h before flow. ^2^Columns were incubated for 7 days before flow. ^3^Percentage of beads/section of slow or fast group. ^4^Fast-corrosion = beads lost more than 1% of their weight. ^5^Slow-corrosion = beads lost less than 1% of their weight. *Only one set of abiotic column control results are shown here. Bold values indicate significant data and Average corrosion.

### Corrosion Rate Distributions of Sulfate-Reducing Bacteria in Multiport Flow Test Column

**FIGURE 2:**
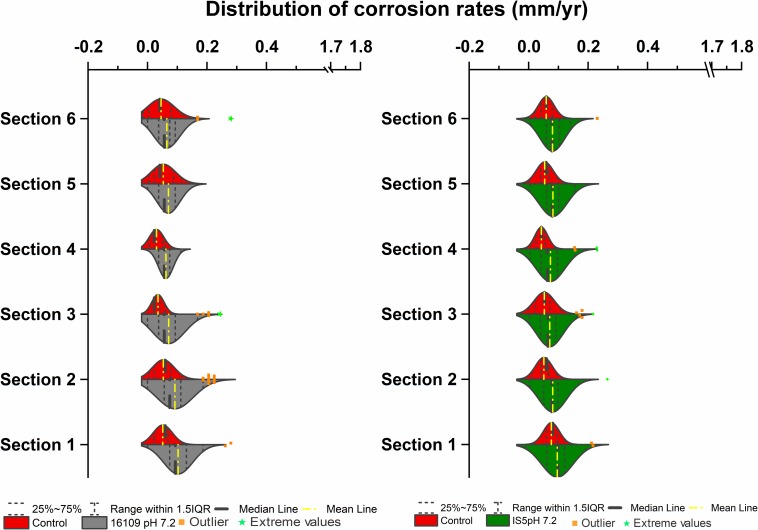
Corrosion rates distribution (mm/yr) of *D. alaskensis* 16109 **(left)** and *D. ferrophilus* **(right)** at pH 7.2 compared against abiotic control in flow-through system columns incubated for 3.5 days without flow. Columns were filled with glass bead as separation medium and operated for 14 days with flow. Corrosion rate distribution, median and outliers of three biological replicates are indicated.

To compare the effect of flow on the corrosion rates of SRB, two sets of columns were established for *D. alaskensis* 16109 and *D. ferrophilus* IS5. Each column section contained 20 uniform carbon steel beads, and the sections were separated using glass beads of similar size. A set of abiotic control was established separately from the three biological replicates. Average general corrosion rates (CR_avg_) of control for *D. alaskensis* 16109 were low, ranging from 0.05 to 0.06 mm/yr between sections 1 to 6 (Table 1 and Supplementary Table S1). The standard deviations were also low (0.036 to 0.059 mm/yr), indicating even corrosion rate (Figure 2, Table 1, and Supplementary Table S1). *D. alaskensis* 16109 had relatively even CR_avg_ between the different sections (Figure 2) as well, which were 0.1 ± 0.05 mm/yr (section 1), 0.01 ± 0.06 mm/yr (section 2), 0.08 ± 0.06 mm/yr (section 3), 0.07 ± 0.03 mm/yr (section 4), 0.07 ± 0.04 mm/yr (section 5) and 0.08 ± 0.04 mm/yr (section 6). Not all sections of *D. alaskensis* 16109 were statistically different from the control (sections 5 and 6), indicating abiotic corrosion unrelated to microbial activities. Sulfide production was observed in the columns (data now shown), maximum sulfide measured was 1.9 mM.

A separate set of flow test columns was established for *D. ferrophilus* IS5. Similar to the previous set of abiotic controls, the CR_avg_ ranged between 0.04 and 0.08 mm/yr, with minimal standard deviations between the different sections of the columns. Similar trend was observed with *D. ferrophilus* IS5, the highest CR_avg_ was observed at the bottom of the column (0.1 ± 0.05 mm/yr; section 1), all other sections had similar corrosion rates between 0.07 and 0.08 mm/yr (Figure 2, right). Statistically, all sections of *D. ferrophilus* IS5 were significantly different than the abiotic control despite the low corrosion rates (Student’s *t*-test, Origin 2019, OriginLab, United States). Sulfide production was detected throughout the columns (data not shown), though the concentrations varied between different sections with a maximum of 0.98 mM. Iron coupon removed from the bottom section of the column showed a cluster of cells on the top layer of the corrosion product (Figure 5).

To further verify MIC by identifying local corrosion, beads were clustered based on their final weights. Beads were considered as fast corroding, if greater than 1% of their initial weight was corroded away. And they are considered as slow corroding if less than 1% of their initial weight was lost. Based on this assumption, all beads per section per treatment were grouped. For abiotic control, slow-corroding beads dominated all sections of the column (80–100%; Supplementary Table S1). With *D. alaskensis* 16109, section 1 (injection site) of the column having the highest number of fast-corroding beads (28%), which decreased to 8% at the effluent region (Supplementary Table S1). In comparison, *D. ferrophilus* IS5 had higher proportion of fast-corroding beads (13–35%) throughout the column (Supplementary Table S1). The highest number of fast-corroding beads was located at the injection region (35%), and the lowest in section 3 (13%). Interestingly, at the top section (section 6), 20% of the beads were still fast corroding, as opposed to the control (15%) and *D. alaskensis* 16109 (8%). In addition to the clustering of beads, a differential corrosion factor (DCF) was introduced (Pinnock et al., 2018), to compare corrosion rates of fast-corroding beads with slow-corroding beads. The highest DCF (CR_fast_/CR_slow_) for *D. alaskensis* 16109 and *D. ferrophilus* IS5 was 2.9 (Supplementary Table S1), which is lower than that of the abiotic control (3.6; Supplementary Table S1). Overall, the general corrosion rates between the two types of SRB were comparable. Comparing with the stationary cultures, the corrosion rate of *D. alaskensis* was tripled (section 1 only; doubled for other sections) while *D. ferrophilus* decreased by 3.6-fold.

### Corrosion Rate Distribution of Methanogenic Archaea in Multiport Flow Test Column

**FIGURE 3:**
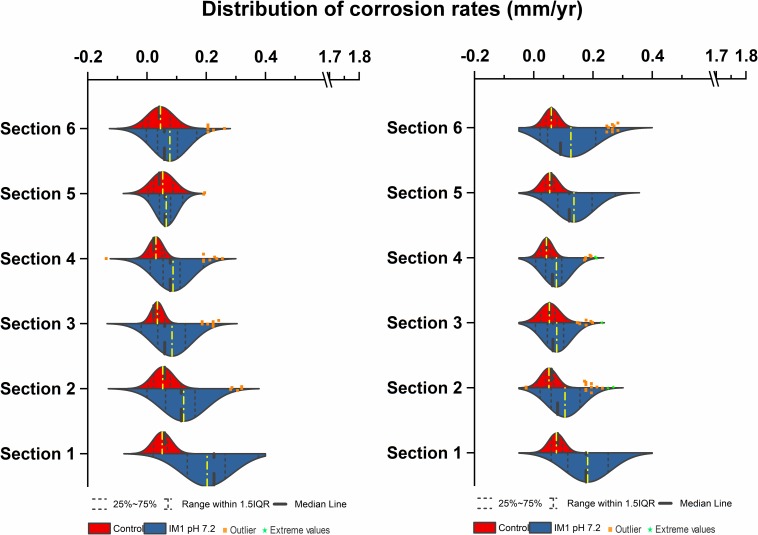
Corrosion rates distribution (mm/yr) of *Methanobacterium* IM1 set 1 **(left)** and *Methanobacterium* IM1 set 2 **(right)** at pH 7.2 compared against abiotic control in flow-through system columns incubated for 3.5 days without flow. Columns were filled with glass bead as separation medium and operated for 14 days with flow. Corrosion rate distribution, median and outliers of three biological replicates are indicated.

**FIGURE 4:**
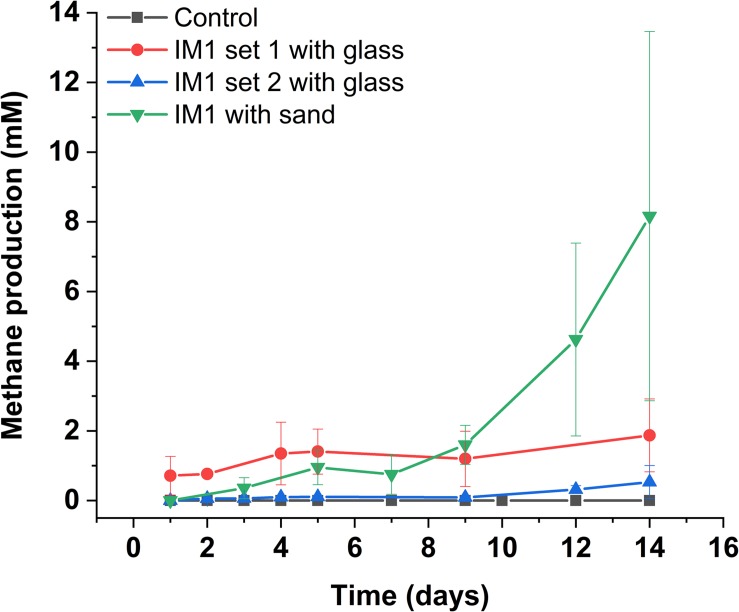
Methane production of *Methanobacterium* IM1 in flow-through system. Glass-packed *Methanobacterium* IM1 columns (set 1 and 2) are compared against sand-packed *Methanobacterium* IM1 column, each data point is the average of three replicates ± SD.

**FIGURE 5:**
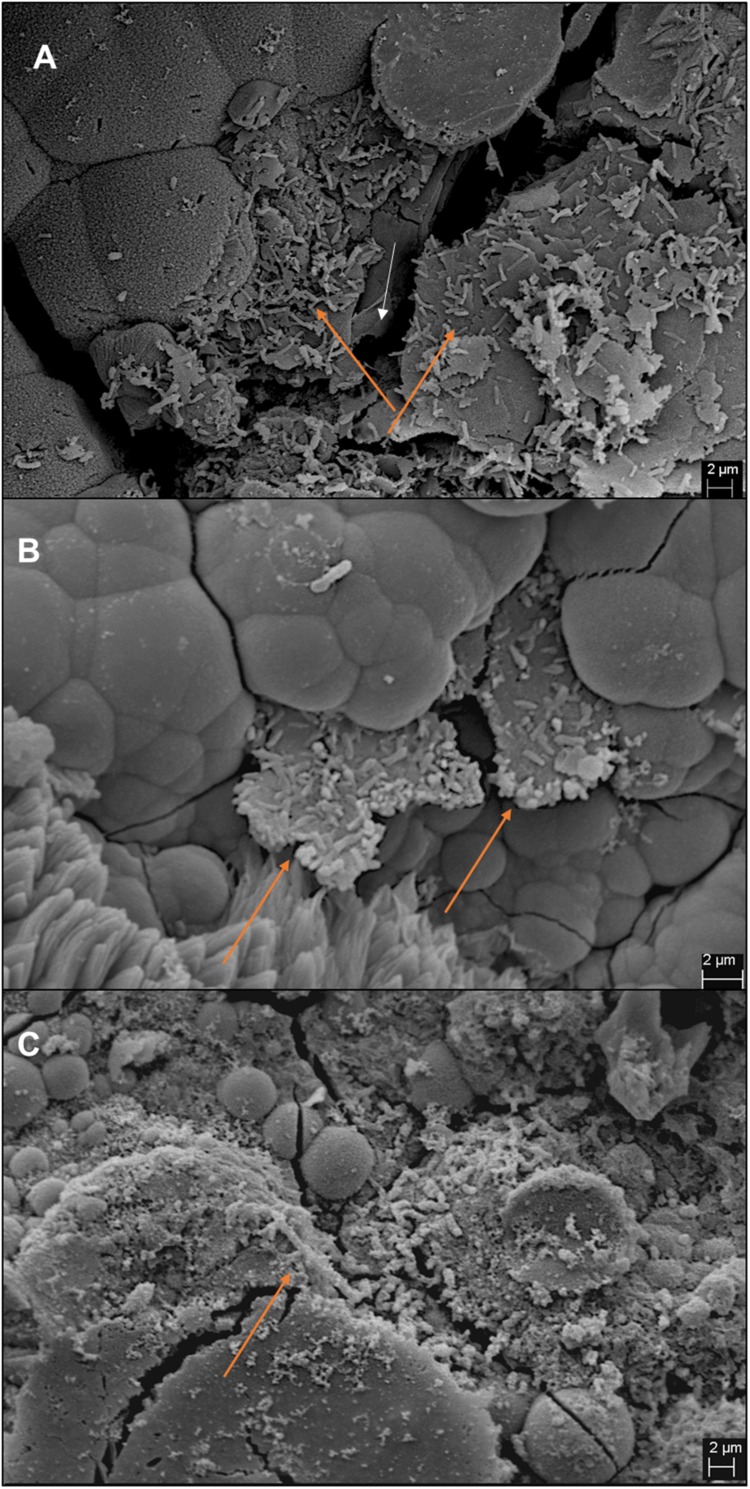
Images of iron coupons extracted from bottom section of the column post-experiment. Coupons from IM1-incubated sand column **(A)**, IM1-incubated glass column **(B)**, and IS5-incubated glass column **(C)** are shown. Cell clusters are indicated by orange arrows and iron surface is indicated by white arrow.

**FIGURE 6:**
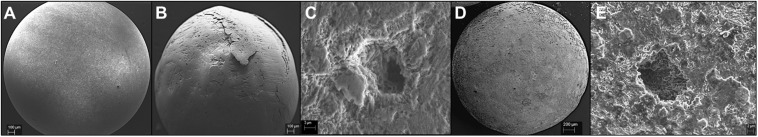
IM1-treated carbon steel beads extracted from the column post-experiment. Beads with 0.52 mm/yr corrosion rate **(B,C)** and 0.38 mm/yr corrosion rate **(D,E)** are shown in comparison to the control bead with no corrosion **(A)**.

In comparison, two separate sets of multiport flow test columns were established to compare corrosion rates of *Methanobacterium* IM1 with SRB. Both sets of the columns were triplicated and ran with two independent incubations to assess reproducibility. Corrosion rates of *Methanobacterium* IM1 set 1 had corrosion rates of 0.2 ± 0.09 mm/yr (section 1), 0.1 ± 0.08 mm/yr (section 2), 0.08 ± 0.07 mm/yr (section 3), 0.09 ± 0.07 mm/yr (section 4), 0.06 ± 0.04 (section 5) and 0.09 ± 0.06 mm/yr (section 6). The maximum corrosion rate (bead with the highest corrosion rate) of *Methanobacterium* IM1 was 0.38 mm/yr (Figures 3, 6D,E), which is higher than that of *D. alaskensis* 16109 (0.28 mm/yr; Figure 2), and control (0.12 mm/yr; Figure 3). Severe pitting was observed on the surface of corroded bead compared to the control (Figures 6A,D,E). The highest DCF (CR_fast_/CR_slow_) was observed at the bottom of the column (4.1; Table 1 and Supplementary Table S1), where 88% of the beads were classified as fast corroding. Interestingly, the proportion of fast-corroding beads decreased over the distance of the column for *Methanobacterium* IM1 (88 to 13%; Supplementary Table S1). This was further reflected on the corrosion rates, where majority of the beads had corrosion rates around 0.2 mm/yr at the bottom of the column and shifted to 0.07 mm/yr on the top (Figure 3, left). After 14 days of operation, the maximum methane production was 1.9 ± 1.0 mM (Figure 4). Interestingly, SEM image of the metal coupon showed cells clustered on sheets that are extending in between large corrosion products (Figure 5B).

Corrosion rates of *Methanobacterium* IM1 set 2 showed highest CR_avg_ at the bottom of the column (0.17 ± 0.08 mm/yr; section 1) as well. The CR_avg_ decreased to 0.06 mm/yr in sections 3 and 4 and increased to 0.11 mm/yr in sections 5 and 6 (Supplementary Table S1). After 14 days of operation, the maximum methane production was 0.65 ± 0.35 mM (Figure 4). All sections of *Methanobacterium* IM1 were significantly different from the control, with a maximum corrosion rate of 0.38 mm/yr, which is 41% higher than the most corroded bead of *D. ferrophilus* IS5 (Figures 2, 3). At the influent region of the column, DCF was 3.6 and 78% of the beads were fast corroding (Table 1). High number of fast corroders was observed throughout the column for *Methanobacterium* IM1 set 2 (15–78%; Figure 3). Furthermore, the corrosion rates of beads did not cluster, as observed in set 1 (Figure 3) but were rather distributed (Figure 3). Part of the beads had corrosion rates above 0.2 mm/yr and the other quartile were around 0.1 mm/yr (Figure 3). In addition, section 5 and 6 of this set also had higher corrosion rates and were dominated by 50% of fast-corroding beads.

### Real Environment Simulation Using Multiport Pipeline

**FIGURE 7:**
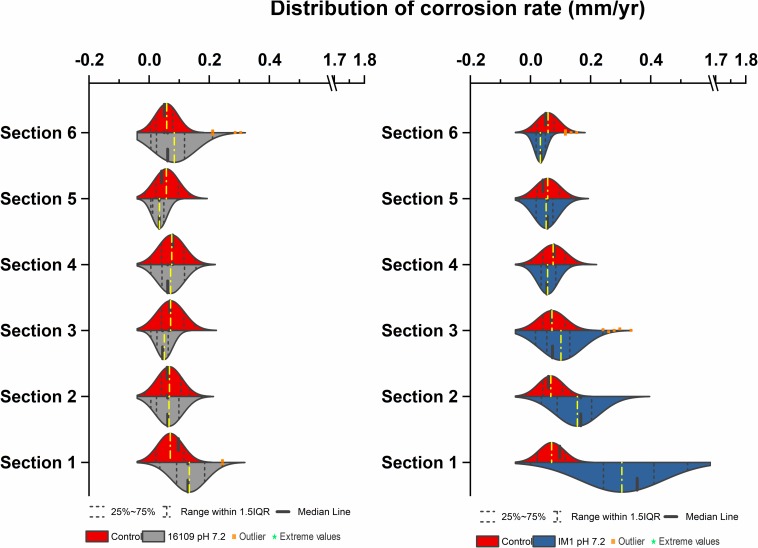
Corrosion rates distribution (mm/yr) of *D. alaskensis* 16109 **(left)** and *Methanobacterium* IM1 **(right)** at pH 7.2 compared against abiotic control in flow system columns incubated for 7 days without flow with sand as a filling medium and operated for 14 days. Corrosion rate distribution, medium and outliers of three biological replicates are indicated.

To test conditions simulating natural sediments, sand was tested as filling material. Higher corrosion rates were observed in sand-packed columns compared to glass-packed columns (Figures 2, 3, 7). Under sand-packed conditions, the average corrosion rates of control ranged between 0.05 and 0.07 mm/yr (Supplementary Table S1). In comparison, the average corrosion rate of *Methanobacterium* IM1 in section 1 was 0.31 ± 0.14 mm/yr and a maximum of 0.52 mm/yr (Figures 6B,C, 7, right). Coupons extracted from section 1 of the *Methanobacterium* IM1 column showed microbial colonization close to the metal surface (Figure 5A) and pitting corrosion on the surface of the corroded beads (Figures 6B,C). The average *Methanobacterium* IM1 corrosion rates decreased over the distance of the column (Figure 7). At the top of the column, CR_avg_ was only 0.04 ± 0.02 mm/yr (section 6; Figure 7), which is 7.75-folds less than section 1. The corrosion rates for all sections of the *Methanobacterium* IM1 columns were statistically significantly different from the abiotic control (*p* < 0.05, two-sample *t*-test, Origin 2019, OriginLab, United States). After 14 days of operation, the maximum methane measured was 8.2 ± 5.3 mM (Figure 4). The highest DCF of sand packed *Methanobacterium* IM1 column was observed at the bottom section of the column (6.4; Table 1 and Supplementary Table S1), which decreased to 0 in section 6. Interestingly, sections 1 (83%) and 2 (62%) of *Methanobacterium* IM1 columns were primarily dominated by fast-corroding beads (Supplementary Table S1), which decreased to 28% in section 3, then 5% in section 4 and reaching 0% in section 6 (Supplementary Table S1). The decrease in the number of fast-corroding beads and increase in slow-corroding beads indicate localized corrosion by *Methanobacterium* IM1, primarily at the injection site.

For *D. alaskensis* 16109, the corrosion rates were relatively even throughout the column (Figure 7, left). The CR_avg_ were 0.1 ± 0.06 mm/yr (section 1), 0.06 ± 0.05 mm/yr (section 2), 0.05 ± 0.03 mm/yr (section 3), 0.07 ± 0.05 mm/yr (section 4), 0.04 ± 0.03 mm/yr (section 5) and 0.08 ± 0.09 mm/yr (section 6; Figure 7). All sections except for section 3 were statistically significantly different from the control (*p* < 0.05, two-sample *t*-test, Origin 2019, OriginLab, United States). The CR of the most-corroded region in *D. alaskensis* 16109-treated columns (CR_avg_ = 0.13 ± 0.063 mm/yr) were significantly lower than that of *Methanobacterium* IM1 (CR_avg_ = 0.31 ± 0.14 mm/yr; *t-*value = 9.15, DF = 99, *p-*value = 7.95e-15; Student’s *t*-test, Origin 2019, OriginLab, United States). Interestingly, sections 5 and 6 had higher DCF (4.2) than section 1 (2.3), though section 1 was dominated by 55% of fast-corroding beads (Supplementary Table S1). Overall, *Methanobacterium* IM1 showed very high corrosion rates in sand-packed systems and more uneven CR distribution than *D. alaskensis* 16109.

## Discussion

Our flow system proved to be an effective corrosion monitoring tool by combining methanogen-induced corrosion with statistical analyses, resulting in high corrosion potential that is relevant for industry. Currently, the two most commonly used methods to measure MIC are electrochemical or static incubations using metal coupons. Despite the ease of operation, both methods are often static and can only replicate static industrial environments, i.e., closed storage tanks, neglecting all scenarios involving environmental fluctuations. Determination of corrosion rates for environmental samples is often conducted by incubating certain sizes and types of metal coupons in an anaerobic serum bottle. After certain incubation period, the differences in weight are used for corrosion rate calculation (Dexter et al., 1991; Park et al., 2011; Enning et al., 2012; Mand et al., 2016). The corrosion rates determined through this method are general corrosion rates, although pit depths can be measured on the coupons but regional corrosion rate is difficult to measure (Voordouw et al., 2016). To accurately determine a clear distribution of corrosion rates, uniform carbon steel ball bearings were introduced (Voordouw et al., 2016). The ball bearings are produced using industrial grade material, i.e., 1010 carbon steel, with high degree of similarity between each specimen (Voordouw et al., 2016). Based on our measurements, the weight between each ball bearing vary between 2.63 × 10^–5^g or 0.026 mg, an ideal candidate to measure corrosion rate distribution. The surface area to volume ratio for the ball bearings are like that of metal coupons, for example the surface to volume ratio of a 2.38 mm diameter bead has a surface area to volume ratio of 25, which is higher than 23, the surface area to volume ratio of a standard sized iron coupon (2 cm × 1 cm × 0.1 cm). However, the experimental procedure is significantly simplified due to the uniformity of the shape and size of the beads, allowing a large number of substrates to be analyzed statistically.

Flow-cell experiments for MIC have been reported numerous times previously for SRB (Stipaničev et al., 2013; Duncan et al., 2014; Pinnock et al., 2018). Most SRB flow cell experiments showed significant pitting corrosion or uneven corrosion rate distribution (Pinnock et al., 2018). However, it remains unclear which region of the flow cell has the highest corrosion as many of the studies reported do not distinguish corrosion rates spatially. Based on our results, average corrosion rates of *D. alaskensis* 16109 and *D. ferrophilus* IS5 were similar between the different sections of the column (Figures 2, 7). The maximum DCF (CR_fast_/CR_slow_) (Pinnock et al., 2018) calculated for *D. alaskensis* 16109 was 2.0 and 2.3 (section 1; Supplementary Table S1) for glass-packed and sand-packed columns, respectively. For *D. ferrophilus* IS5, this factor was 2.2 (section 1; Supplementary Table S1). Despite the differences in corrosion mechanism (Enning and Garrelfs, 2014; Jia et al., 2019), the corrosion factor and corrosion rates were comparable between the two SRB strains. One explanation for the low corrosion rates of SRB, is the availability of sulfate and passivation of FeS. Coupons extracted from *D. ferrophilus* columns showed SRB cells colonized the top layer of the coupon (Figure 5), where sulfate availability is the highest. Analyzes on a corroded pipe extracted from an oil field showed similar results, where high numbers of SRB were found on the top layer (Larsen et al., 2010). The lower DCF observed for SRB than abiotic control could be due to the formation of a passivating FeS layer, which can be caused by the low concentrations of measured sulfide (<1.9 mM). Several studies indicated the formation of a thin FeS layer will have a passivating effect on iron by reducing the rate of dissolution (Newman et al., 1991; Lee et al., 1995; Enning and Garrelfs, 2014). However, investigations on the corrosion products of SRB at different regions of the column are needed. Overall, high concentrations of sulfate at stationary conditions prove to be the ideal growth condition for SRB. Whereas, in low-sulfate flow conditions, substrate availability, mechanical stress and porosity are limiting factors for SRB cells.

On the contrary, iron-utilizing methanogenic cells clustered closely to the iron surface, since they are strictly dependent upon the evolution H_2_ on the metal surface (Larsen et al., 2010). The same pipe cut which showed an abundant number of SRB on the top layer, further demonstrated a high number of archaea cells close to the metal surface, creating a cellular gradient between methanogens and SRB (Larsen et al., 2010). Several corrosive strains of methanogens are classified in the family of *Methanococcaceae* (Mori et al., 2010; Tsurumaru et al., 2018), including *M. maripaludis*, which are known hydrogenotrophic methanogens that utilize elemental iron for methanogenesis through H_2_ formation (Tsurumaru et al., 2018). Members of the *M. maripaludis* such as KA1 and OS7 are capable of secreting extracellular hydrogenases that transfers electrons from the metal surface to form hydrogen for methanogenesis. However, it is known that *Methanobacterium* IM1 have higher affinity for elemental iron than for H_2_ (Beese-Vasbender et al., 2015; Kip et al., 2017; in ‘t Zandt et al., 2019). Though the specific mechanisms are still under investigation, it was speculated that *Methanobacterium* IM1 can tightly adhere to the metal surface, allowing cell-surface redox mediators to effectively transfer electrons (Beese-Vasbender et al., 2015). Currently, methanogens are defined as ‘corrosion protectors,’ with corrosion rates ranging between 0.02 and 0.065 mm/yr (Daniels et al., 1987; Mori et al., 2010; Liang et al., 2014; Mand et al., 2014; Okoro C. et al., 2016; Okoro C.C. et al., 2016) and production of non-conductive carbonate-based products, i.e., siderite (Mori et al., 2010; Kip et al., 2017; Barker et al., 2018) (Eq. 4). Under static conditions, the buildup of abiotic and biotic corrosion products, particularly siderite is more easily achieved than under flowing conditions (Barker et al., 2018). According to Eq. 5, the restriction to electron access is more likely under static conditions, explaining the reported lower corrosion rates. Under flow conditions, the average corrosion rates of *Methanobacterium* IM1 range between 0.2 and 0.3 mm/yr, with maximum corrosion rates above 0.5 mm/yr, which is 1.3 to 3 times higher than the corrosion rates obtained under static conditions (0.15 mm/yr; Table 1), and up to 25 times higher than reported methanogenic-corrosion rates. The highest corrosion rates of *Methanobacterium* IM1 were observed often at the incubation site (sections 1 and 2). The decreased corrosion rates in sections 4–6 could be explained with the build of corrosion products, which were carried away from the influent region. But additional investigations on the local conditions, i.e., redox potential and water chemistry, using microsensors are needed. Under environmental-fluctuating conditions, i.e., marine sediments or pipeline, scale precipitation and ion dissolution are dependent upon flow velocity. In one abiotic study, fluid turbulence caused formation of localized corrosion (Han et al., 2010), but the eventual buildup of siderite due to mass-transfer limits may slow down corrosion (Han et al., 2010). But it was shown that biofilm can influence mass-transfer, particularly for pitting corrosion (Taherzadeh et al., 2012; Xu et al., 2016). However, the corrosion products of *Methanobacterium* IM1 need further investigation, particularly at the different regions of the flow system. Overall, increased corrosion rates under flow conditions indicate enhanced growth for methanogens with high iron affinity.

Interestingly, increased average general corrosion rates were observed using sand as packing material compared to glass beads. Despite the similarity in pore volume between the two types of packing materials, corrosion rates in sand-packed columns were higher than that of glass packed columns, particularly for *Methanobacterium* IM1. The average corrosion rates of *Methanobacterium* IM1 section 1 in sand packed columns were 1.5 times than that of glass columns. Compared to glass beads, sand grains are more textured and denser, allowing enhanced microbial attachment. Furthermore, sand grains enhances the growth of microorganisms, it has been found that each sand grain can habit 10^5^ cells (Probandt et al., 2018) with only 0.5 μm between each cell (Probandt et al., 2018). Sand grains can shield microorganism from mechanical sheer stress, while providing necessary porosity for substrate availability (Probandt et al., 2018). A comparison between incubation time was additionally conducted, where an incubation of 7 days, selected based on previous bioreactor studies (Callbeck et al., 2011; An et al., 2017; Chen et al., 2017), was compared with 3.5 days and 24 h. Results showed a linear relationship between incubation time and corrosion rate (Supplementary Figure S3), indicating a possible role of biofilm in the corrosion mechanism of methanogens that can be further amplified in sand-packed environments. Additionally, sand grains can further mimic environmental conditions in marine sediments, soil and oil reservoir. Currently, sand-packed reactors were used to investigate oil reservoir related studies, such as souring control and enhanced oil recovery (Gieg et al., 2011; Gassara et al., 2015; An et al., 2017; Suri et al., 2019) targeting SRB and nitrate-reducing bacteria. Bioreactor or studies under flow regarding methanogens have been largely neglected due to the difficulties maintaining an anaerobic system that allows methane measurements. However, to evaluate the potential of methanogen-induced MIC, certain environmental conditions must be established, for example *Methanobacterium* IM1 was originally isolated from the marine sediment. Though the evolutionary history of the strain remains unclear but based on our current results *Methanobacterium* IM1 has higher iron-utilizing efficiency in sand compared to static and glass-packed environments.

## Conclusion

We have shown that standardized flow-tests should be incorporated as part of regular MIC monitoring, especially for methanogen-induced MIC. Different types of packing material, flow rates, influent solution, i.e., field water samples, and metals, offer great flexibility while providing a comprehensive corrosion profile. To further our understanding of microbial activities at the interface between cells and metal surface, microsensors and additional environmental parameters need to be applied, i.e., biocides. To increase the resolution of spatial-corrosion profiles, longer columns made of different materials can be used. Overall, by standardizing flow systems as part of regular laboratory experiments allows more accurate MIC measurements and development of effective mitigation strategies.

## Data Availability Statement

All datasets generated for this study are included in the article/Supplementary Material.

## Author Contributions

BA was responsible for experimental setup, data collection, interpretation, execution of the experiments, drafting and revision of the manuscript. SK worked on the setup, conducted the experiments for stationary cultures and drafted the manuscript. OO provided conception and input of the work, edited and reviewed of the manuscript. AK provided supervision, the conception of the work and final approval of the manuscript version to be published.

## Conflict of Interest

The authors declare that the research was conducted in the absence of any commercial or financial relationships that could be construed as a potential conflict of interest.
