# Supplementary material for: Iron to Gas: Versatile Multiport Flow-Column Revealed Extremely High Corrosion Potential by Methanogen-Induced Microbiologically Influenced Corrosion (Mi-MIC)
Source: Front Microbiol. 2020 Mar 31;11:527. doi: 10.3389/fmicb.2020.00527 (PMC7136402; doi:10.3389/fmicb.2020.00527)
Supplement: Supplementary file 1 [file Data_Sheet_1.docx]

Supplementary Material

# Supplementary Data

**Supplementary**

**Table S1** Complete comparison of corrosion rates and distribution between flow-through system sections and stationary cultures. Sulfate reducing bacteria (*D. alaskensis* and *D. ferrophilus* IS5) are compared against methanogen (*Methanobacterium* IM1).

|  | **Flow system** | | | | | | | | | | | | | | | | **Stationary cultures** | | | |
| --- | --- | --- | --- | --- | --- | --- | --- | --- | --- | --- | --- | --- | --- | --- | --- | --- | --- | --- | --- | --- |
|  | Column section | Control* | 16109^1^ | | IS5^1^ | | IM1^1^  set 1 | | IM1^1^  set 2 | | Control | | 16109^2^ | | IM1^2^ | | Control | 16109 | IS5 | IM1 |
| Total fast (%) | 1 | 10 | 28 | | 35 | | 88 | | 78 | | 30 | | 55 | | 83 | | N/A | | | |
|  | 2 | 15 | 23 | | 22 | | 57 | | 38 | | 25 | | 13 | | 62 | |  |  |  |  |
|  | 3 | 0 | 18 | | 13 | | 30 | | 18 | | 30 | | 3 | | 28 | |  |  |  |  |
|  | 4 | 5 | 3 | | 18 | | 28 | | 15 | | 35 | | 25 | | 5 | |  |  |  |  |
|  | 5 | 20 | 13 | | 23 | | 13 | | 57 | | 15 | | 3 | | 7 | |  |  |  |  |
|  | 6 | 15 | 8 | | 20 | | 27 | | 45 | | 15 | | 23 | | 0 | |  |  |  |  |
| CR_Fast_ (mm/yr) | 1 | 0.12 | 0.2 | | 0.15 | | 0.22 | | 0.21 | | 0.09 | | 0.17 | | 0.36 | |  |  |  |  |
|  | 2 | 0.12 | 0.2 | | 0.15 | | 0.18 | | 0.17 | | 0.10 | | 0.14 | | 0.20 | |  |  |  |  |
|  | 3 | 0.00 | 0.2 | | 0.15 | | 0.17 | | 0.16 | | 0.10 | | 0.12 | | 0.19 | |  |  |  |  |
|  | 4 | 0.10 | 0.1 | | 0.15 | | 0.17 | | 0.18 | | 0.10 | | 0.14 | | 0.12 | |  |  |  |  |
|  | 5 | 0.12 | 0.1 | | 0.15 | | 0.14 | | 0.18 | | 0.10 | | 0.14 | | 0.14 | |  |  |  |  |
|  | 6 | 0.12 | 0.2 | | 0.13 | | 0.17 | | 0.21 | | 0.10 | | 0.20 | | 0.00 | |  |  |  |  |
| Total Slow (% ) | 1 | 90 | 72 | | 65 | | 12 | | 22 | | 70 | | 45 | | 17 | |  |  |  |  |
|  | 2 | 85 | 77 | | 78 | | 43 | | 62 | | 75 | | 88 | | 38 | |  |  |  |  |
|  | 3 | 100 | 82 | | 87 | | 70 | | 82 | | 68 | | 98 | | 72 | |  |  |  |  |
|  | 4 | 90 | 97 | | 82 | | 72 | | 85 | | 65 | | 75 | | 95 | |  |  |  |  |
|  | 5 | 80 | 87 | | 77 | | 87 | | 43 | | 85 | | 98 | | 93 | |  |  |  |  |
|  | 6 | 85 | 92 | | 80 | | 73 | | 55 | | 85 | | 78 | | 100 | |  |  |  |  |
| CRSlow (mm/yr) | 1 | 0.04 | 0.1 | | 0.07 | | 0.05 | | 0.06 | | 0.05 | | 0.07 | | 0.06 | |  |  |  |  |
|  | 2 | 0.04 | 0.1 | | 0.06 | | 0.05 | | 0.04 | | 0.05 | | 0.05 | | 0.07 | |  |  |  |  |
|  | 3 | 0.03 | 0.1 | | 0.05 | | 0.04 | | 0.03 | | 0.05 | | 0.05 | | 0.06 | |  |  |  |  |
|  | 4 | 0.03 | 0.1 | | 0.05 | | 0.05 | | 0.04 | | 0.05 | | 0.05 | | 0.05 | |  |  |  |  |
|  | 5 | 0.04 | 0.1 | | 0.06 | | 0.05 | | 0.04 | | 0.05 | | 0.03 | | 0.04 | |  |  |  |  |
|  | 6 | 0.03 | 0.1 | | 0.07 | | 0.05 | | 0.03 | | 0.05 | | 0.05 | | 0.04 | |  |  |  |  |
| CR_Fast_/CR_Slow_ | 1 | 2.6 | 2.0 | | 2.2 | | 4.1 | | 3.6 | | 1.8 | | 2.3 | | 6.4 | |  |  |  |  |
|  | 2 | 2.7 | 2.9 | | 2.4 | | 3.3 | | 4.1 | | 1.9 | | 2.7 | | 3.0 | |  |  |  |  |
|  | 3 | 0.0 | 2.8 | | 2.9 | | 3.9 | | 4.8 | | 2.1 | | 2.4 | | 3.1 | |  |  |  |  |
|  | 4 | 3.6 | 2.1 | | 2.8 | | 3.1 | | 4.6 | | 2.1 | | 2.9 | | 2.4 | |  |  |  |  |
|  | 5 | 3.1 | 2.2 | | 2.5 | | 2.9 | | 4.0 | | 2.3 | | 4.2 | | 3.1 | |  |  |  |  |
|  | 6 | 3.5 | 2.7 | | 2.0 | | 3.2 | | 6.4 | | 2.2 | | 4.3 | | 0.0 | |  |  |  |  |
| CR average | 1 | 0.05 | 0.10 | | 0.10 | | 0.20 | | 0.17 | | 0.06 | | 0.13 | | 0.31 | | 0.022 | 0.034 | 0.35 | 0.15 |
|  | 2 | 0.05 | 0.10 | | 0.08 | | 0.13 | | 0.09 | | 0.06 | | 0.06 | | 0.15 | |  |  |  |  |
|  | 3 | 0.03 | 0.08 | | 0.07 | | 0.08 | | 0.06 | | 0.06 | | 0.05 | | 0.10 | |  |  |  |  |
|  | 4 | 0.03 | 0.07 | | 0.07 | | 0.09 | | 0.06 | | 0.07 | | 0.07 | | 0.05 | |  |  |  |  |
|  | 5 | 0.05 | 0.07 | | 0.08 | | 0.06 | | 0.12 | | 0.05 | | 0.04 | | 0.05 | |  |  |  |  |
|  | 6 | 0.05 | 0.08 | | 0.08 | | 0.09 | | 0.11 | | 0.05 | | 0.08 | | 0.04 | |  |  |  |  |
| Fast-corrosion= beads lost more than 1% of total weight | | | | | | | | | | | | | | | | | | | | |
| Slow-corrosion=beads lost less than 1% of total weight | | | | | | | | | | | | | | | | | | | | |
|  |  |  | |  | |  | |  | |  | |  | |  | |  |  |  |  |  |


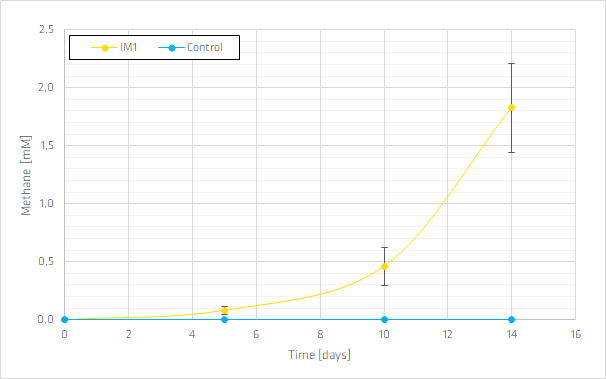


Figure S1: Methane production [mM] by *Methanobacterium* IM1 (yellow) compared to the abiotic control (blue) over 14 days. Error bars indicate the standard deviation of three replicates (IM1) or duplicates (control).


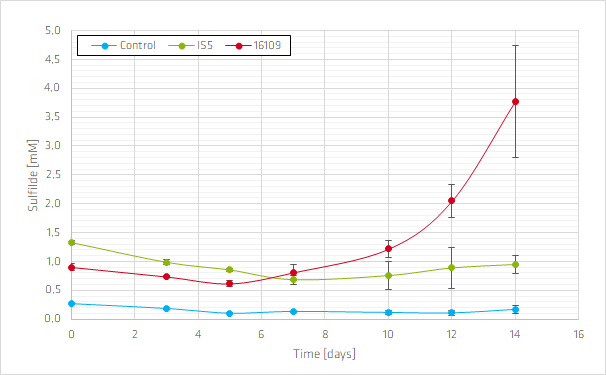


Figure S2: Formation of hydrogen sulfide [mM] by *D. ferrophilus* IS5 (green) and *D. alaskensis* 16109 (red) compared to the abiotic control (blue) over 14 days. Error bars indicate the standard deviation of three replicates (IS5 and 16109) or duplicates (control).

Figure S3: Correlation between incubation days and average corrosion of section 1 for *Methanobacterium* IM1
